# Nano/Micro Hybrid Bamboo Fibrous Preforms for Robust Biodegradable Fiber Reinforced Plastics

**DOI:** 10.3390/polym13040636

**Published:** 2021-02-20

**Authors:** Junsik Bang, Hyunju Lee, Yemi Yang, Jung-Kwon Oh, Hyo Won Kwak

**Affiliations:** 1 Department of Agriculture, Forestry and Bioresources, College of Agriculture & Life Sciences, Seoul National Universtiy, 1 Gwanak-ro, Gwanak-gu, Seoul 08826, Korea; bangsik.e@snu.ac.kr (J.B.); leehj3030@naver.com (H.L.); yemi98@snu.ac.kr (Y.Y.); jungoh@snu.ac.kr (J.-K.O.); 2 Research Institute of Agriculture and Life Sciences, Seoul National University, 1 Gwanak-ro, Gwanak-gu, Seoul 08826, Korea

**Keywords:** bamboo fiber, cellulose nanofiber, adhesion, multiscale hybridization, fibrous composite

## Abstract

The focus on high-strength and functional natural fiber-based composite materials is growing as interest in developing eco-friendly plastics and sustainable materials increases. An eco-friendly fibrous composite with excellent mechanical properties was prepared by applying the bamboo-derived nano and microfiber multiscale hybridization phenomenon. As a result, the cellulose nanofibers simultaneously coated the micro-bamboo fiber surface and adhered between them. The multiscale hybrid phenomenon implemented between bamboo nano and microfibers improved the tensile strength, elongation, Young’s modulus, and toughness of the fibrous composite. The enhancement of the fibrous preform mechanical properties also affected the reinforcement of biodegradable fiber-reinforced plastic (FRP). This eco-friendly nano/micro fibrous preform can be extensively utilized in reinforced preforms for FRPs and other green plastic industry applications.

## 1. Introduction

Problems related to petroleum resource depletion and the potential dangers of microplastics are continuously raised, and the need for eco-friendly materials continues to rise. The consumption of reusable or biodegradable natural polymers is essential, especially when taking raw materials into account. Since ancient times, natural fibers, such as wool, silk, and cotton, have been used in clothing, and cellulose, which is a wood-based polymer, has been actively employed in the paper industry. In addition, since the inception of FRPs, studies using natural fibers as reinforcing agents have been repeatedly reported.

Natural fibers are not only abundant and inexpensive, but are also biodegradable due to their nature-derived properties. Therefore, researchers have examined their application in FRP reinforcement [1]. However, the hydrophilic properties of natural fiber reduce compatibility with synthetic polymer matrices. Chemical modification of natural fiber surfaces has been attempted to improve the compatibility between natural fibers andmatrix polymers [2]. Although, this surface modification process mostly inhibits the economical and environmentally-friendly properties of natural fibers.

Nanocellulose is a nanomaterial obtained from wood and agricultural byproducts [3,4,5,6], as well as from bacteria [7,8], and has a diameter numbering tens of nanometers [9]. This nanomaterial has low density, high strength, and excellent transparency [10]. As a result, research is being performed to utilize nanocellulose in various applications ranging from food packaging materials, composite-material nanofillers, electronic devices, and biomaterials [11]. Due to the nanomorphology of nanocellulose and the resulting strong mechanical strength, composite materials with various general-purpose polymers, such as polyolefin and polyester [9], are also being developed [12]. In addition, as biodegradable polyesters such as polylactic acid (PLA), polycaprolactone (PCL), and polybutylene succinate (PBS) were developed, studies to apply nanocellulose to compensate for the insufficient mechanical strength were also actively conducted [7]. However, nanocellulose also has hydrophilic properties similar to natural fibers and wood-based biomass, which are their raw materials. Thus, insufficient compatibility with a polymer matrix is considered a limitation [13]. Biomimetic methods have been tried to overcome this drawback. When bacteria biosynthesized nanocellulose, a water-soluble polymer, such as hydroxyethyl cellulose or polyvinyl alcohol, was introduced to prepare a nanocellulose/polymer composite [7,14]. These biomimetic nanocellulose/polymer composites showed higher tensile properties than composites prepared by simple mixing [12]. However, this method has the disadvantage of interfering with the cellulose biosynthesis of bacteria, and mass production is impossible [15,16].

Recently, attempts have been made to use nanocellulose as a "green" binder in the manufacture of preforms for hierarchical composites. Lee et al. used bacterial cellulose as a binder to prepare sisal fiber preforms for the reinforcement of polyacrylated epoxidized soybean oil (AESO) [16]. As a result, it is evident that the mechanical properties of the preform improved significantly. The mechanical properties of poly AESO/sisal preforms manufactured by thermal curing following vacuum-assisted resin injection are also extensively improved. It is known that the excellent binding and adhesion ability of these nanocelluloses can be clearly expressed in natural fibers, including sisal and silk fibers [17,18]. However, most of the research results indicate that bacterial cellulose was used as a binder [19], and there has been no effort to manufacture a hierarchical fibrous-composite preform using microfibers and nanocellulose produced from the same tree species.

Bamboo is a plant resource that is cultivated worldwide [20], especially in Asia and South America. Since ancient times, bamboo has been steadily used in furniture and construction materials due to its rigid mechanical properties [20]. In addition, as bamboo fiber extraction technology advances, attempts to use it as a sustainable raw material for high-performance composite materials and advanced carbon materials are steadily progressing. [21,22,23]. Furthermore, various types of nanocellulose manufacturing technologies utilize bamboo fibers as raw materials [24,25,26]. Therefore, in the development of eco-friendly composite materials, it is very attractive to develop a facile preforming process using the hierarchical nano/micro morphology of bamboo fiber [27,28].

This study’s primary purpose is to fabricate a fibrous composite preform with improved mechanical properties through a multiscale, hybridizing process using bamboo-derived microfibers and cellulose nanofibers. First, the optimal preparation conditions (temperature and time) were established to minimize the energy consumption required to manufacture the fibrous composite preform. The physicochemical properties of the prepared micro/nano bamboo-fibrous composite preform were analyzed according to the nanocellulose content, and the changes in mechanical properties were confirmed accordingly. Finally, FRP was prepared using PBS, which is an actual biodegradable polyester, and changes in mechanical properties of FRP due to the reinforcing effect of nano/microfiber preforms were observed.

## 2. Materials and Methods

### 2.1. Materials

The bamboo fiber (BF) used in the experiment was purchased from Studio Buntu (Seoul, Korea) and cut into 2 cm lengths; a bamboo cellulose nanofiber (CNF) dispersion (1 wt.%) was purchased from Chuetsu Pulp & Paper Co., Ltd. (Toyama, Japan); Direct Red 80 (analytical grade, dye content, 25%) was purchased from Sigma Aldrich (Yongin, Korea); and polybutylene succinate (PBS) pellets (BG5000M, 100% PBS) were kindly provided by Anko Bioplastics (Wonju, Korea).

### 2.2. Preparation of BF/CNFs

The bamboo fiber and CNF composite were readied using a thermocompression method. First, 2 g of bamboo fiber was immersed in 50 mL of a CNF dispersion solution of different concentrations (0–2 w∙v^−1^ %), stirred sufficiently, and then adequately dehydrated by hand in a 3 × 10 cm frame. Afterward, the BF/CNF mixture was thermocompressed with a mini hot press (QM900S, Qmasys, Uiwang, Korea). BF/CNF preforms were prepared by varying the compression temperatures at 25 °C, 60 °C, 80 °C, 120 °C, 160 °C, and 180 °C to find the optimum thermocompression temperature. Moreover, BF/CNF preforms were produced with different compression times for 1, 2, 3, 5, 10, and 15 min to optimally establish the thermocompression time. The thermocompression pressure in all experiments was fixed at 20 MPa. BF/CNFs with different CNF contents were denoted as BF/CNFn, where n denotes the wt.% of CNFs based on BF weight. Ten samples of BF/CNFs were prepared through single preforming process and used for tensile testing and characterization.

### 2.3. Preparation of BF/CNFs Preform Reinforced PBS Composite

In preparing the FRP sample, a bamboo fiber preform and a PBS pellet were directly impregnated using a hot press. At this time, the process temperature was set at 120 °C, and the pressure was fixed at 5 MPa. The PBS pellet and bamboo fiber preform were put in a ratio of 5:5 in reference to the total weight. The FRP composites were named PBS-BF/CNFn. Subsequently, distilled water was used to wash off unabsorbed dye molecules present in the BF/CNF, and an image of the BF/CNF preform was obtained.

### 2.4. Characterization of BF/CNFs

Optical images of BF and CNF were obtained using a Galaxy Note 10 (Samsung, Seoul, Korea). The morphology of the BFs was observed through a Field emission scanning electron microscope (FE-SEM, SUPRA 55VP, Carl Zeiss, Oberkochen, Germany), and the morphology of the CNFs was detected using a Field emission transmission electron microscope (FE-TEM, JEM2100F, JEOL, Tokyo, Japan). The thickness of the BF/CNF preform was measured using a thickness measuring device (CD-20CP, Mitutoyo, Kawasaki, Japan). The preform’s moisture content was measured using a moisture meter (MB120, OHAUS, New Jersey, United States). The BF/CNF preform was immersed in 60 mL of Direct Red 80 dye solution (0.0025 wt.%) and incubated for 30 min to visualize the distribution of CNFs within the BF nonwoven structure. Next, distilled water was applied to remove the unreacted dye molecules from the sample until no red color was observed on the paper towel, which was pressed onto the BF/CNF fibrous composite sample.

The BF/CNF preform’s physical structure was confirmed by measuring the porosity, and the absolute density was determined by He pycnometry (AccuPyc 1330, Micromeritics, Georgia, United States). Samples were weighed before being loaded into the measuring chamber. The nonwoven preform’s apparent density (ρe) was calculated from the mass and sheath volume of the nonwoven preform. The porosity of the preform was computed with the following Equation (1):

(1) $$Porosity\;\left(\%\right)=\;\left\{\frac{1-Enveloped\;density}{Absolute\;Density}\right\}\;\times 100$$

The morphological properties of the BF/CNF preform were observed through FE-SEM. A pull-to-break experiment was performed using a universal testing machine (34SC-1, Instron, Massachusetts, United States) to evaluate the BF/CNF preform’s mechanical properties. A preform sample with a width of 10 mm, a length of 20 mm, and a thickness of 0.8 mm was utilized. A 1 kN load cell was applied, and the experiment was carried out at a tensile speed of 0.5 mm∙min^−1^. The experiment was repeated five times to obtain the average value and standard deviation of tensile strength, modulus of elasticity, elongation, and toughness. The PBS-BF/CNF FRP mechanical properties were measured through a tensile test, and the experiment was performed under the same conditions as the BF/CNF preform.

## 3. Results

### 3.1. Morphology of Bamboo Fibers and Bamboo-Derived Nanocellulose

**Figure 1:**
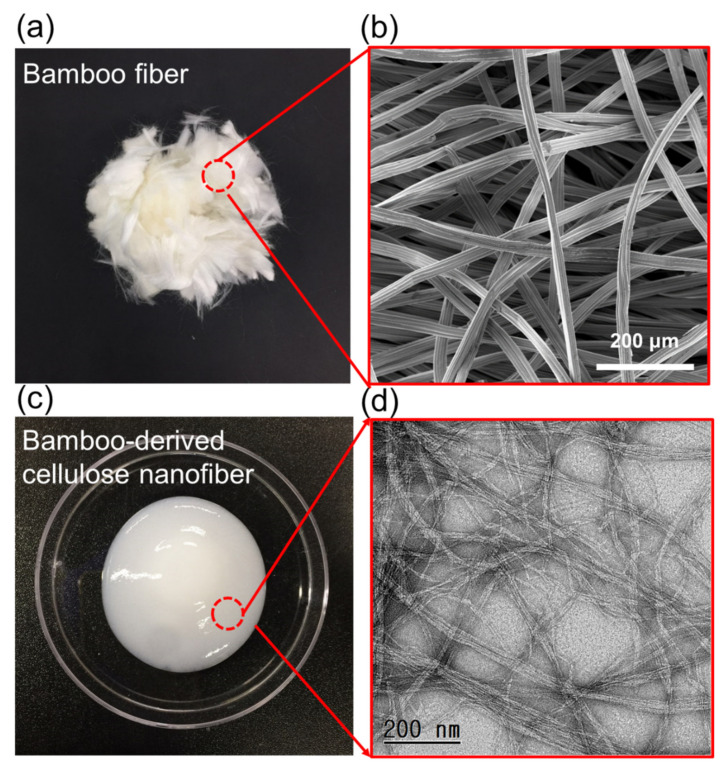
Morphology of bamboo fibers (BFs) and cellulose nanofibrils (CNFs). (**a**) Optical and (**b**) FE-SEM images of BFs. (**c**) Optical and (**d**) FE-TEM images of CNFs.

Figure 1 displays an optical image and electron microscope image of BF and its derived cellulose nanofibrils (CNFs). The single microfiber consists of many elementary fibrils in bamboo fibers, and rough and flat oval-shaped fibers can be observed. The rough surface of these bamboo fibers is known to cause the mechanical reinforcement effect of FRP by increasing the interfacial active bonding sites between the fiber surface and the polymer matrix. However, the effect is insignificant due to the roughness on the micrometer scale and the extreme hydrophilicity difference between the matrix (hydrophobic polymer) and filler (hydrophilic fiber) [29]. On the other hand, bamboo nanofibers are dispersed in water and have viscosity like a sticky glue substance. In addition, it can be seen from the transmission electron microscope image that it is composed of nanofibers with a diameter of about 10–20 nm. The viscous flow of CNFs means that the aqueous CNFs can act as a general adhesive binder and penetrate the fiber surface [17,30]. Additionally, the CNF’s long aspect ratio with nanofibrillar morphology is expected to enable the CNFs to impart a nano brush structure to the BF fiber surface in the BF/CNF system and enable bonding between the BF fibers.

### 3.2. Optimization of the Preform Bamboo Fibrous Composite Process

**Figure 2:**
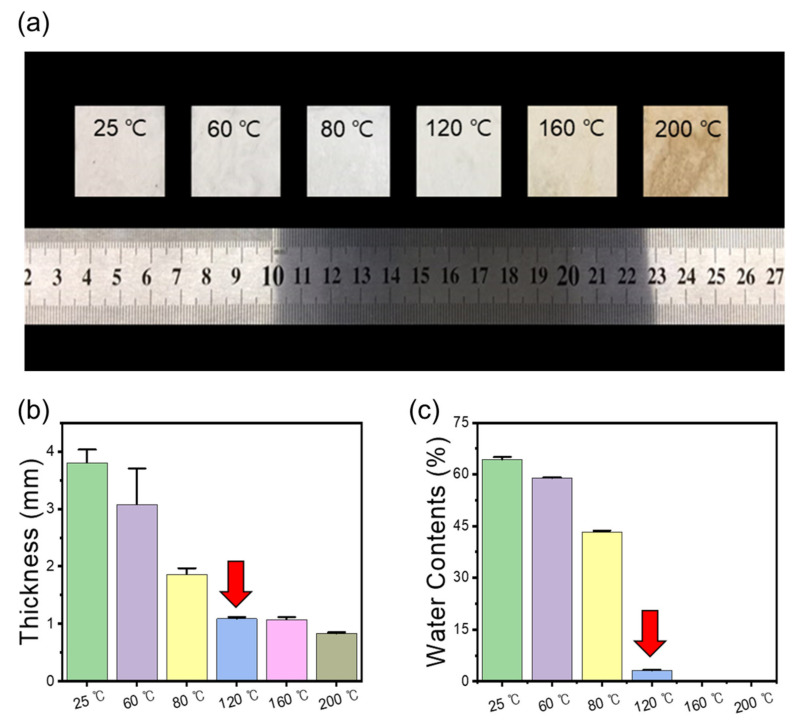
Effect of thermocompressing temperature on the physical properties of BF/CNF fibrous preforms. (**a**) Optical images, (**b**) thickness, and (**c**) water contents of BF/CNF fibrous preforms.

Manufacture of preform-type fiber reinforcement not only minimizes separation due to the difference in compatibility between the fiber and the matrix polymer, but also makes it possible to easily manufacture fiber-reinforced plastic materials through methods such as laminating and impregnation of polymer melt [31,32]. When preparing a fibrous composite preform using nanocellulose, the manufacturing process proceeds to wet the CNF aqueous solution to microfibers, dehydration, and thermocompression processes. Among these processes, thermocompression greatly influences the thickness and moisture content of the fiber preform. In addition, to design energy-efficient polymer processing, it is necessary to optimize the heat treatment temperature and compression time in the thermocompression process. To find the optimum preforming condition for the rigid fibrous preform along with an effective dehydration process, the physical properties of the BF/CNFs determined according to the thermocompression temperature were confirmed through the thickness and moisture content (Figure 2). As seen from the figure, the color change of BF/CNF did not occur significantly until heat treatment at 120 °C, whereas yellowing occurred when thermal compression was performed at high temperatures of 160 °C and 200 °C. This yellowing phenomenon of the BF/CNF preform due to the high heat treatment temperature was caused by thermal degradation of cellulose [33]. The more severe the yellowing, the more degradation proceeded [34]. Since sufficient dehydration does not occur in the low temperature condition of 20–80 °C, the thickness of the preform is thick and not uniform, which means that it does not provide sufficient structural stability when used as a reinforcing preform for fiber reinforced plastics. As the heat treatment temperature increases, the thickness and water content of the BF/CNF decreases, meaning that heat treatment at a sufficient temperature is required for dehydration of the absorbed water molecules in the wetting process. Through a sufficient dehydration process, the gap between BF and CNFs becomes close, which means that various types of interactions, including hydrogen bonds between nano and microfibers, can easily occur. In summarizing the above results, it was found that 120 °C could be an optimal temperature condition necessary to minimize the preform’s moisture content while preventing thermal decomposition of both BFs and CNFs.

**Figure 3:**
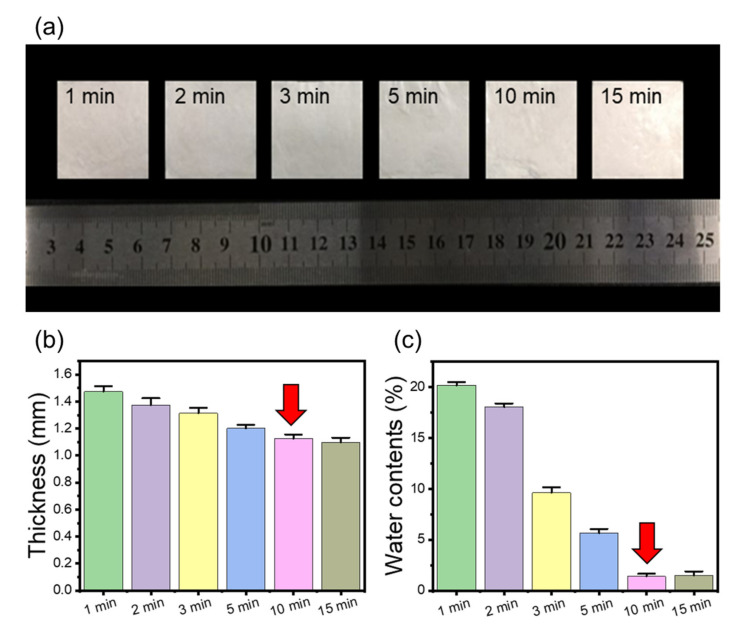
Effect of thermocompression time on the physical properties of BF/CNF fibrous preforms. (**a**) Optical images, (**b**) thickness, and (**c**) water contents of BF/CNF fibrous preforms.

In fibrous preform manufacturing, the pressing time is a crucial factor along with the pressing temperature. The preform was manufactured by varying the thermocompression time from 0 to 15 min at 120 °C to establish an energy-friendly manufacturing process by minimizing the time while ensuring the BF/CNF composite’s structural stability. Figure 3 depicts the thickness and moisture content results of the BF/CNF preform according to the thermocompression time. The optimum temperature is confirmed as 120 °C because the color change does not appear during the 15-min thermocompression process. As the thermal compression time lengthens, the moisture content and thickness of the preform fall because the water molecules that participate in the wetting process of BF/CNF evaporate. This drop in moisture content and thickness decreases continuously until a thermal compression time of 10 min when equilibrium is reached. Compared with the initial BF/CNF preforms, which only proceeded with 1-min thermal compression (1.47 mm thickness and 21% moisture content), the preforms that underwent 10 min of thermal compression reduced the moisture content to 1.8%. The thickness was reduced to 1.18 mm because the BF/CNFs could penetrate the evaporated water space. Taken together, the optimal condition for minimizing energy consumption, and ensuring sufficient dehydration and a rigid preform structure, is thermal compression bonding at 120 °C for 10 min.

### 3.3. Effect of CNF Addition on the Physical Properties of BF/CNF Fibrous Preforms

**Figure 4:**
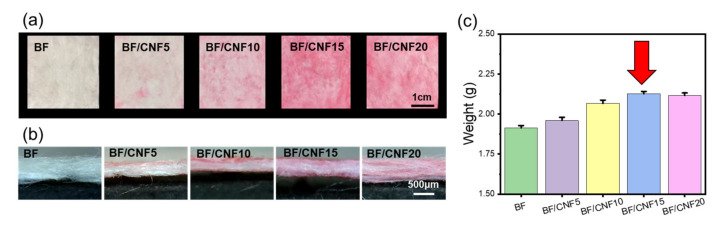
Effect of CNF addition on the visual inspection of BF/CNF fibrous preforms. (**a**,**b**) Optical images of Direct Red 80 dyed BF/CNF fibrous preforms, and (**c**) weight-gain ratio results.

It is essential to deposit a nanomaterial on a microfibrous structure when preparing a nano/micro hybrid fibrous preform. The nanomaterial deposition process can be primarily carried out through impregnation and spraying methods [35]. Separate equipment is required for the spraying method, and penetration into the fiber structure may be challenging. BF/CNFs hybrid preforms can be constructed through a dehydration technique after thoroughly mixing and stirring the CNF aqueous solution and BF in the immersion method. This immersion mixing method also has the advantage of being able to easily control the added CNFs in the BF/CNF framework by varying the concentration of aqueous CNFs. However, a sufficient number of CNFs may not penetrate the BF nonwoven structure when a larger volume of aqueous CNFs is added over the microfiber’s absorption properties or when high-viscosity CNFs are input according to a high concentration. Figure 4 illustrates the dyeing images and weight results of BF/CNF fibrous preforms prepared by varying the content of CNFs. Even with the same bamboo cellulose, there is a difference in dyeing kinetics depending on the fiber morphology. For micrometer-scale BFs, an adequate dyeing process does not undergo because the dye molecules cannot be adsorbed on the BF microfiber’s surface and diffuse into the interior in a short dyeing time of 10 min [36]. On the other hand, for nanoscale CNFs with a high surface area of the nanofibrillar morphology, the dye molecules easily and rapidly adsorb onto the CNF surface, and the color remains satisfactory even after the washing process.

This difference in dyeing properties between BFs and CNFs made it possible to confirm the distribution of CNFs present in the BF/CNF preform. As shown in Figure 4, as the content of CNFs increased to 15 wt.%, the red color of the BF/CNFs gradually became darker, meaning that more CNFs were present in the BF/CNFs. On the other hand, when 20 wt.% CNFs were included, there was no significant difference in color from BF/CNF15, implying that more than 15 wt.% of CNFs can no longer participate in BF fiber coating and fiber-to-fiber bonding; these CNFs are separated in the dehydration process. In addition, the difference in dyeability of the BF/CNFs preform according to the amount of CNFs added was consistent with the actual weight increase result.This pattern was similar to the results of manufacturing silk fiber/CNF fibrous preforms [37]. Altogether, the amount of CNFs in the BF/CNF preform can be easily controlled by varying the concentration of aqueous CNF solutions.

**Figure 5:**
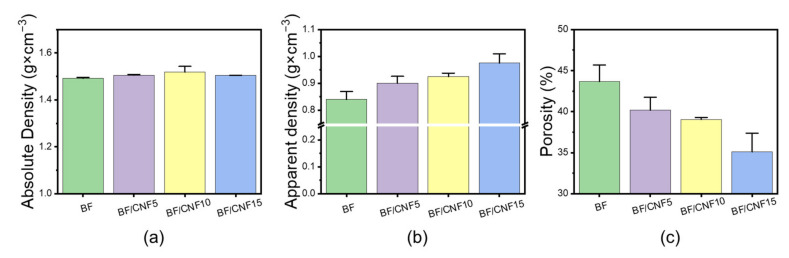
Effect of CNF addition level on the physical properties of BF/CNF fibrous preforms. (**a**) Absolute density, (**b**) apparent density, and (**c**) calculated porosity.

When CNFs are added to the BF fiber nonwoven fabric manufacturing process, the nanofibrils coat the microfibers, and the interfacial bonding between the microfibers is more strongly induced [38]. Since CNFs have a higher surface area (14.5 m^2^∙g^−1^) than wood pulp (1.8 m^2^∙g^−1^) [17], they can be expected to act as a sufficient binder even with a small amount of CNFs. Furthermore, among various nanocellulose morphologies, nanofibrillated CNFs with a high aspect ratio could be more effective in bonding BF microfibers than road-like cellulose nanocrystals (CNCs). The absolute and apparent densities were measured to examine the effect of CNF binder addition on the BF/CNF fibrous preform physical properties (Figure 5). Even when CNFs were added, the absolute density did not change significantly because both BFs and CNFs were composed of the same cellulose. However, the apparent density is calculated by including the pore volume of materials, so the volume increases as the CNF rises. The quantity of CNFs grows because CNFs are located in the void structure of the BF nonwoven structure. This rise in the apparent density and the resulting fall in porosity have a high correlation. The BF nonwoven fabric has the highest porosity of 43.4%, but when the CNFs were included as a binding material, the coating and interfibrillar bonding of CNFs onto the BF nonwoven structure were able to occur; therefore, BF/CNF15 has the lowest porosity of 35.4%.

**Figure 6:**
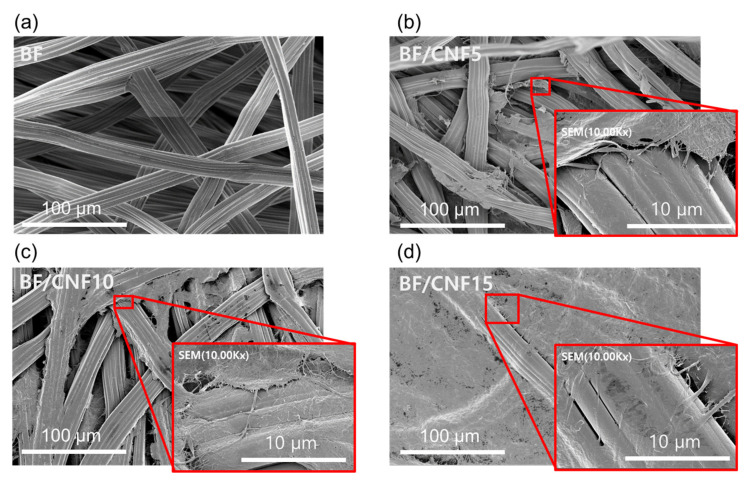
FE-SEM images of BF/CNF fibrous preforms with various amounts of CNFs. The insets represent high-magnitude images. (**a**) BF, (**b**) BF/CNF5, (**c**) BF/CNF10, and (**d**) BF/CNF15.

To investigate the effect of CNF addition on the morphology of the BF/CNF fibrous preform, FE-SEM observations were conducted, and the corresponding images are presented in Figure 6. In the case of nonwoven BF, no change in fiber diameter and morphology was observed despite its thermocompression. It has a porous, nonwoven structure, and the fibers are connected smoothly. This means that the previously selected preforming conditions (120 °C for 10 min) do not cause physical damage to the preform. For BF/CNF5, the CNF bundle is coated on the surface of the BF fiber. More CNF-coated BFs were observed for BF/CNF10, and CNF bundles began to distribute in the interfibrillar space of the preform structure. CNF bundles cover the entire surface of the BF/CNF preform in BF/CNF15, and the pore structure is significantly lost. Based on the morphology findings, the addition of CNFs increases BF fiber coating and the interfacial bonding between microfibers.

### 3.4. Effect of CNF Addition on the Mechanical Properties of BF/CNF Fibrous Preforms

**Figure 7:**
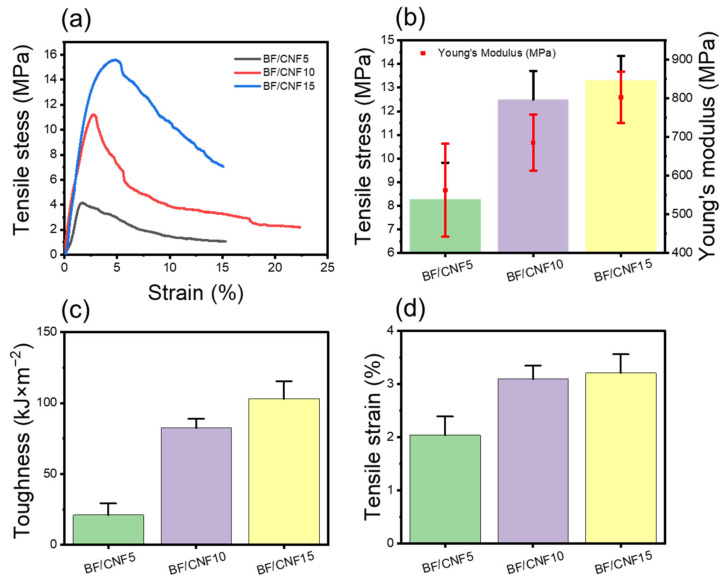
Mechanical properties of BF/CNF fibrous preforms. (**a**) Representative tensile stress–strain curve, (**b**) tensile stress and Young’s modulus, (**c**) toughness, and (**d**) tensile strain.

The fibrous preform’s mechanical properties are closely related to the final FRP product manufactured as a reinforcing agent. Figure 7 summarizes the tensile properties of the fabricated BF/CNF nonwoven preforms. The tensile test results could not be obtained for BF nonwoven samples without CNFs because the fibers loosened as soon as the tension load was applied, and the nonwoven structure was damaged. Alternatively, a tensile test was possible for BF/CNF5. As a result, it was determined that the tensile strength was 8.1 MPa, and Young’s modulus was 570 MPa. CNFs begin to act as coatings and binders as they penetrate the BF nonwoven structure. The mechanical reinforcing effect of CNFs increased as the CNF quantity rose. BF/CNF15 demonstrated a tensile strength of 13.8 MPa, and Young’s modulus of 770 MPa. In addition, as the number of CNFs increased, the tensile elongation of the BF/CNF preform also grew. As CNFs are input, the breakage of BF/CNFs is converted from BF-oriented brittle fracture behavior to elastic and progressive failure due to the energy dissipation effect of the CNF bundle. Vilchez prepared chicken hair fiber/CNF preforms and confirmed that as the quantity of CNFs enlarged, progressive failure appeared, increasing tensile elongation [18]. This progressive failure behavior of the BF/CNFs by CNFs consequently contributed to a substantial surge in the BF/CNF preform toughness.

### 3.5. Mechanical Properties of BF/CNF Preform Reinforced PBS Composites

**Figure 8:**
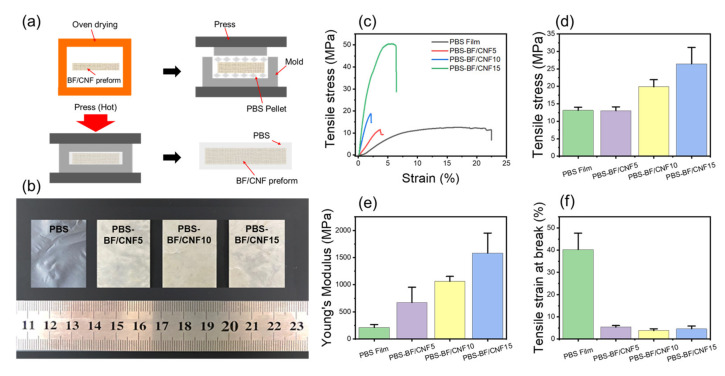
Mechanical properties of BF/CNF reinforced PBS composites. (**a**) Schematic of the PBS-BF/CNF composite fabrication process, (**b**) optical images of PBS-BF/CNF composites, and (**c**–**f**) tensile properties of PBS-BF/CNF composites.

Polybutylene succinate (PBS) was used as a biodegradable polymer matrix to examine the effect of the physical properties of BF/CNF preform on the mechanical properties of the final biodegradable FRP. PBS is known to be biodegradable in a composting environment and using natural succinic acid as a monomer maximizes its valorization [39,40,41]. Since PBS has a lower melting temperature (~120 °C) than PLA and Polypropylene (PP), it has the advantage of preventing the thermal decomposition of natural fiber preforms when manufacturing FRP composites [2,39,42]. Figure 8a displays a direct impregnation process for PBS-BF/CNF FRP composite preparation considering cost-efficiency [31]. As shown in Figure 8b, the PBS-BF/CNF FRP composite exhibited a color similar to the BF/CNF preform, implying that the preform did not undergo thermal decomposition and subsequent yellowing during the direct PBS impregnation process. Figure 8c shows the representative stress–strain curves of the PBS-BF/CNF FRP composite tested under uniaxial tension. PBS mainly exhibited elastic deformation behavior, low tensile strength, and Young’s modulus of 13.4 MPa and 270 MPa, respectively. When the BF/CNF preform was used as a reinforcing agent [43,44], the FRP composite modulus began to increase rapidly, and this phenomenon was evident in BF/CNF15 with its high CNF content. As a result, PBS-BF/CNF15 improves the tensile strength by 240% (26.4 MPa), and Young’s modulus by 700% (1.6 GPa), compared to PBS. The improved mechanical properties mean that the BF fiber’s properties do not merely exhibit the BF/CNF preform’s reinforcing effect, but the improved mechanical properties of nano/micro fibrous preforms directly participate in the reinforcing effect of FRP [35]. In addition, the nanobrush surface morphology formed on the CNFs coated on BF could maximize the interaction with the PBS matrix [19,45,46].

## 4. Conclusions

In this study, using a hybridization process of bamboo-derived nano/microfibers, a green and mechanically robust fibrous preform for fiber-reinforced plastics was successfully fabricated. The optimal thermocompression environment for minimizing fiber thermal degradation and obtaining a dense fibrous preform was 120 °C and 10-min compression conditions. As CNF was introduced into the BF/CNF preforming process, the structural stability of the fibrous preform was significantly enhanced. The hybrid phenomenon of BF/CNFs provided a nano-brush morphology that maximized the interaction with the matrix polymer on the surface of the fibrous preform and, at the same time, increased the tight bonding between the BF fibers, making the internal structure of the preform more compact. Ultimately, even when the BF/CNFs preform was used as an eco-friendly reinforcing material in biodegradable fiber reinforced plastic (PBS-BF/CNFs composites), the improved mechanical properties of the fibrous preforms could be effectively expressed. As a result, the PBS-BF/CBF15 composite material improved the tensile strength by 240% (26.4 MPa) and Young’s modulus by 700% (1.6 GP) compared to the neat PBS plastics. The synthetic binder or crosslinker-free and energy-efficient micro/nano hybridized green fibrous preforms manufacturing process has excellent potential to be widely utilized in the field of eco-friendly biodegradable fiber-reinforced composite materials in the future.

## Data Availability

The data presented in this study are available on request from the corresponding author.

## References

[1] Chin S.C., Tee K.F., Tong F.S., Ong H.R., Gimbun J. (2020). Thermal and mechanical properties of bamboo fiber reinforced composites. Mater. Today Commun..

[2] Han Q., Zhao L., Lin P., Zhu Z., Nie K., Yang F., Wang L. (2020). Poly(butylene succinate) biocomposite modified by amino functionalized ramie fiber fabric towards exceptional mechanical performance and biodegradability. React. Funct. Polym..

[3] García A., Gandini A., Labidi J., Belgacem N., Bras J. (2016). Industrial and crop wastes: A new source for nanocellulose biorefinery. Ind. Crops Prod..

[4] Jonoobi M., Mathew A.P., Oksman K. (2012). Producing low-cost cellulose nanofiber from sludge as new source of raw materials. Ind. Crops Prod..

[5] Moslemi A., Zolfagharlou koohi M., Behzad T., Pizzi A. (2020). Addition of cellulose nanofibers extracted from rice straw to urea formaldehyde resin; effect on the adhesive characteristics and medium density fiberboard properties. Int. J. Adhes. Adhes..

[6] Tibolla H., Pelissari F.M., Rodrigues M.I., Menegalli F.C. (2017). Cellulose nanofibers produced from banana peel by enzymatic treatment: Study of process conditions. Ind. Crops Prod..

[7] Fernandes M., Souto A.P., Gama M., Dourado F. (2019). Bacterial Cellulose and Emulsified AESO Biocomposites as an Ecological Alternative to Leather. Nanomaterials.

[8] Lee K.Y., Buldum G., Mantalaris A., Bismarck A. (2014). More than meets the eye in bacterial cellulose: Biosynthesis, bioprocessing, and applications in advanced fiber composites. Macromol. Biosci..

[9] Hao W., Wang M., Zhou F., Luo H., Xie X., Luo F., Cha R. (2020). A review on nanocellulose as a lightweight filler of polyolefin composites. Carbohydr. Polym..

[10] Siró I., Plackett D. (2010). Microfibrillated cellulose and new nanocomposite materials: A review. Cellulose.

[11] Chen W., Yu H., Lee S.Y., Wei T., Li J., Fan Z. (2018). Nanocellulose: A promising nanomaterial for advanced electrochemical energy storage. Chem. Soc. Rev..

[12] Wang L., Roach A.W., Gardner D.J., Han Y. (2017). Mechanisms contributing to mechanical property changes in composites of polypropylene reinforced with spray-dried cellulose nanofibrils. Cellulose.

[13] Chu Y., Sun Y., Wu W., Xiao H. (2020). Dispersion Properties of Nanocellulose: A Review. Carbohydr. Polym..

[14] Neves R.M., Ornaghi H.L., Zattera A.J., Amico S.C. (2021). Recent studies on modified cellulose/nanocellulose epoxy composites: A systematic review. Carbohydr. Polym..

[15] Wang J., Tavakoli J., Tang Y. (2019). Bacterial cellulose production, properties and applications with different culture methods—A review. Carbohydr. Polym..

[16] Moniri M., Boroumand Moghaddam A., Azizi S., Abdul Rahim R., Bin Ariff A., Zuhainis Saad W., Navaderi M., Mohamad R. (2017). Production and Status of Bacterial Cellulose in Biomedical Engineering. Nanomaterials.

[17] Fortea-Verdejo M., Lee K.-Y., Zimmermann T., Bismarck A. (2016). Upgrading flax nonwovens: Nanocellulose as binder to produce rigid and robust flax fibre preforms. Compos. Part A.

[18] Vilchez V., Dieckmann E., Tammelin T., Cheeseman C., Lee K.-Y. (2020). Upcycling Poultry Feathers with (Nano)cellulose: Sustainable Composites Derived from Nonwoven Whole Feather Preforms. ACS Sustain. Chem Eng..

[19] Lee K.-Y., Ho K.K.C., Schlufter K., Bismarck A. (2012). Hierarchical composites reinforced with robust short sisal fibre preforms utilising bacterial cellulose as binder. Compos. Sci. Technol..

[20] Mohammed L., Ansari M.N.M., Pua G., Jawaid M., Islam M.S. (2015). A Review on Natural Fiber Reinforced Polymer Composite and Its Applications. Int. J. Polym. Sci..

[21] Ju Z., Zhan T., Zhang H., He Q., Hong L., Yuan M., Cui J., Cheng L., Lu X. (2020). Strong, Durable, and Aging-Resistant Bamboo Composites Fabricated by Silver In Situ Impregnation. ACS Sustain. Chem. Eng..

[22] Cui R., Pan L., Deng C. (2015). Synthesis of carbon nanocoils on substrates made of plant fibers. Carbon.

[23] Le Phuong H.A., Izzati Ayob N.A., Blanford C.F., Mohammad Rawi N.F., Szekely G. (2019). Nonwoven Membrane Supports from Renewable Resources: Bamboo Fiber Reinforced Poly(Lactic Acid) Composites. ACS Sustain. Chem. Eng..

[24] Abe K., Yano H. (2009). Comparison of the characteristics of cellulose microfibril aggregates isolated from fiber and parenchyma cells of Moso bamboo (Phyllostachys pubescens). Cellulose.

[25] Hu Z., Zhai R., Li J., Zhang Y., Lin J. (2017). Preparation and Characterization of Nanofibrillated Cellulose from Bamboo Fiber via Ultrasonication Assisted by Repulsive Effect. Int. J. Polym. Sci..

[26] Wang H., Zhang X., Jiang Z., Li W., Yu Y. (2015). A comparison study on the preparation of nanocellulose fibrils from fibers and parenchymal cells in bamboo (Phyllostachys pubescens). Ind. Crops Prod..

[27] Borri A., Castori G., Corradi M., Speranzini E. (2014). Durability Analysis for FRP and SRG Composites in Civil Applications. Key Eng. Mater..

[28] Chiu H.-H., Young W.-B. (2020). Characteristic study of bamboo fibers in preforming. J Compos. Mater..

[29] Miao M., Shan M. (2011). Highly aligned flax/polypropylene nonwoven preforms for thermoplastic composites. Compos. Sci. Technol..

[30] Mautner A., Kwaw Y., Weiland K., Mvubu M., Botha A., John M.J., Mtibe A., Siqueira G., Bismarck A. (2019). Natural fibre-nanocellulose composite filters for the removal of heavy metal ions from water. Ind. Crops Prod..

[31] Kim S.H., Park C.H. (2017). Direct impregnation of thermoplastic melt into flax textile reinforcement for semi-structural composite parts. Ind. Crops Prod..

[32] Codispoti R., Oliveira D.V., Olivito R.S., Lourenço P.B., Fangueiro R. (2015). Mechanical performance of natural fiber-reinforced composites for the strengthening of masonry. Compos. Part B.

[33] Manasoglu G., Kanik M., Yildirim K. (2019). Effect of fixation conditions on yellowing behavior of cellulose powder-coated fabrics. J. Eng. Fibers Fabr..

[34] Matsuo M., Umemura K., Kawai S. (2012). Kinetic analysis of color changes in cellulose during heat treatment. J. Wood Sci..

[35] Uribe B.E.B., Soares-Pozzi A.C., Tarpani J.R. (2019). Nanocellulose-coated carbon fibers towards developing hierarchical polymer matrix composites. Mater. Today Proc..

[36] Sribenja S., Saikrasun S. (2015). Adsorption behavior and kinetics of lac dyeing on poly (ethyleneimine)-treated bamboo fibers. Fibers Polym..

[37] Eom J., Park S., Jin H.-J., Kwak H.W. (2020). Multiscale hybridization of natural silk-nanocellulose fibrous composites with exceptional mechanical properties. Front. Mater..

[38] Lu Z., Hu W., Xie F., Hao Y. (2017). Highly improved mechanical strength of aramid paper composite via a bridge of cellulose nanofiber. Cellulose.

[39] Xu J., Guo B.H. (2010). Poly (butylene succinate) and its copolymers: Research, development and industrialization. Biotechnol. J..

[40] Nghiem N., Kleff S., Schwegmann S. (2017). Succinic Acid: Technology Development and Commercialization. Fermentation.

[41] Song H., Lee S.Y. (2006). Production of succinic acid by bacterial fermentation. Enzyme Microb. Technol..

[42] Petchwattana N., Sanetuntikul J., Sriromreun P., Narupai B. (2017). Wood plastic composites prepared from biodegradable poly (butylene succinate) and Burma Padauk sawdust (Pterocarpus macrocarpus): Water absorption kinetics and sunlight exposure investigations. J. Bionic Eng..

[43] Li Y., Jiang L., Xiong C., Peng W. (2015). Effect of Different Surface Treatment for Bamboo Fiber on the Crystallization Behavior and Mechanical Property of Bamboo Fiber/Nanohydroxyapatite/Poly(lactic-co-glycolic) Composite. Ind. Eng. Chem. Res..

[44] Manalo A.C., Wani E., Zukarnain N.A., Karunasena W., Lau K.-t. (2015). Effects of alkali treatment and elevated temperature on the mechanical properties of bamboo fibre–polyester composites. Compos. Part B.

[45] Heng J.Y.Y., Pearse D.F., Thielmann F., Lampke T., Bismarck A. (2012). Methods to determine surface energies of natural fibres: A review. Compos. Interfaces.

[46] Juntaro J., Pommet M., Kalinka G., Mantalaris A., Shaffer M.S.P., Bismarck A. (2008). Creating Hierarchical Structures in Renewable Composites by Attaching Bacterial Cellulose onto Sisal Fibers. Adv. Mater..

